# Enhanced Moisture-Reactive Hydrophilic-PTFE-Based Flexible Humidity Sensor for Real-Time Monitoring

**DOI:** 10.3390/s18030921

**Published:** 2018-03-20

**Authors:** Heekyeong Park, Sungho Lee, Seok Hwan Jeong, Ui Hyun Jung, Kidong Park, Min Goo Lee, Sunkook Kim, Joonhyung Lee

**Affiliations:** 1 School of Advanced Materials Science & Engineering, Sungkyunkwan University, Suwon 16419, Korea; parkpa19@skku.edu (H.P.); csh0122@skku.edu (S.H.J.); juh11@skku.edu (U.H.J.); 2 Korea Electronics Technology Institute, Gyeonggi 13488, Korea; 2sungho@gmail.com (S.L.); emingoo@keti.re.kr (M.G.L.); 3 Division of Electrical and Computer Engineering, Electrical Engineering Building, Louisiana State University, Baton Rouge, LA 70809, USA; kidongp@lsu.edu; 4 Device & System Research Center, Samsung Advanced Institute of Technology, Suwon 443-803, Korea

**Keywords:** flexible humidity sensor, hydrophilic polytetrafluoroethylene (H-PTFE), real-time monitoring

## Abstract

Flexible sensors connected to cell phones are a promising technology that can aid in continuously monitoring signals in our daily lives, such as an individual’s health status and information from buildings, farms, and industry. Among such signals, real-time humidity monitoring is crucial to a comfortable life, as human bodies, plants, and industrial environments require appropriate humidity to be maintained. We propose a hydrophilic polytetrafluoroethylene (H-PTFE)-based flexible humidity sensor integrated with readout circuitry, wireless communication, and a mobile battery. To enhance its sensitivity, linearity, and reliability, treatment with sodium hydroxide implements additional hydroxyl (OH) groups, which further enhance the sensitivity, create a strong linearity with respect to variations in relative humidity, and produce a relatively free hysteresis. Furthermore, to create robust mechanical stability, cyclic upward bending was performed for up to 3000 cycles. The overall electrical and mechanical results demonstrate that the flexible real-time H-PTFE humidity sensor system is suitable for applications such as wearable smart devices.

## 1. Introduction

The development of flexible humidity sensors has been gaining interest in several applications, including industrial production processes, environmental monitoring, and healthcare [1,2,3]. Monitoring and controlling humidity is of considerable importance for the reliable operation of various systems in the industrial and environmental fields. Furthermore, the integration of a humidity sensor with other physiological sensors has drawn attention from the research community and industry owing to its potential to produce diverse applications in the domain of wearable healthcare. Also, the humidity information obtained from sensors is transferred immediately to users via wireless communication [4], and analysis, prediction, and diagnosis can be performed based on the collected data. 

Among several types of humidity sensors, the capacitive humidity sensor has been widely investigated because of its capability to function at high temperatures, fully recover from condensation, and resist chemical vapors [5,6]. Capacitive humidity sensors consist of a hygroscopic dielectric material sandwiched between a pair of electrodes, forming a small capacitor. Most of these capacitive sensors are based on dielectric changes in the sandwiched material upon water vapor uptake as a measure of the water vapor content. The water adsorption occurs by physical hydrogen bonds through the van der Waals interaction of water molecules with the hydrophilic groups of the dielectric material [7].

Many materials have been studied as dielectric materials for capacitive humidity sensors, including polymers, ceramics, and composites [8,9,10]. Ceramics have generally been adopted for humidity sensors because of their low price, excellent thermal properties, mechanical strength, and rapid response [11,12]. However, issues of aging and large hysteresis have limited their potential as monitoring sensors that require long-term operation and high accuracy. In order to achieve long-term use and reliability, as well as high sensitivity and stability, various kinds of polymer-based sensing materials have been considered [13,14]. 

In this report, we present a flexible capacitive humidity sensor using hydrophilic polytetrafluoroethylene (H-PTFE) as a dielectric sensitive film for water uptake. H-PTFE is a polymer-based membrane that has a random porous structure with a 0.45 μm pore size and 80% porosity. Due to its porous structure, H-PTFE has a large volume area and flexibility like a paper. In addition, H-PTFE is known to have thermal stability, chemical stability, and tear resistance, which also provide advantages for monitoring sensors [15]. Furthermore, alkali metals are reported to be effective in etching the polytetrafluoroethylene (PTFE) surface by breaking the very strong carbon–fluorine bonds, which enables PTFE to incorporate hydroxyl (OH) groups into the porous structure [16,17]. In this study, for further improvement of the hydrophilicity of H-PTFE, additional OH groups were added to the H-PTFE surface through a treatment of sodium hydroxide (NaOH). This resulted in a dramatic increase in the capacitance value, approximately by a factor of 2 × 10^4^, with respect to the variation in relative humidity (RH) over a range of 45~90%. In addition, linear behavior of the sensing performance and a low hysteresis were clearly observed. Moreover, the proposed sensor was not significantly sensitive to temperature fluctuations and showed no decrease in performance, even after a cyclic bending test of 3000 cycles. Finally, we implemented a real-time monitoring system through system-level integration of the fabricated flexible H-PTFE humidity sensor with readout circuits and a Bluetooth module. Finally, the proposed system was attached to a curved surface for real-time monitoring of the moisture of the materials, which demonstrates the potential of our sensor as a humidity-monitoring application.

## 2. Materials and Methods

### 2.1. Fabrication of Flexible PTFE Humidity Sensor

The H-PTFE membrane was obtained from Merck Millipore, Burlington, USA. First, the H-PTFE film was prepared with dimensions of 1.5 × 1.5 cm. In order to form the capacitive electrode on the H-PTFE film, a nickel alloy shadow mask (Ambi stencils, Pyeongtaek, Korea) was used for selective deposition of the electrode source. The pattern of the shadow mask was an interdigitated structure with eight fingers. Each finger width was 250 µm and the finger-to-finger distance was 150 µm. The thickness of the shadow mask is 100 μm, which could be adhered completely to the H-PTFE substrate beforemetal deposition. After fixation of the shadow mask above the H-PTFE film, titanium/gold (20 nm/100 nm) was deposited by e-beam evaporator under a vacuum of 10^−6^ Torr. Titanium was used for an adhesion layer in this process. After deposition, the shadow mask was removed. To enhance the mechanical stability of sensors, poly(ethylene terephthalate) (PET) film (70 µm) was laminated beneath the thin H-PTFE film.

### 2.2. NaOH Activation for Enhanced Water Absorption Ability

The fabricated H-PTFE humidity sensor was incubated in NaOH solution (1 M, Samchun, Seoul, Korea) for 2 h at room temperature. This process was performed with stirring. After removing the sensor from the NaOH solution, the sensor was dipped into DI water to rinse it enough, then it was dried by nitrogen gas. Finally, the remaining water in the H-PTFE film was completely removed by annealing in a vacuum oven (VDO-08NG, Samheung Energy, Sejong, Korea) with a temperature of 120 °C for 1 h. 

### 2.3. Characteristics

High-resolution field-emission scanning electron microscopy (FE-SEM) images were acquired using Merlin, Carl Zeiss operating at 10 kV. The optical microscope images were obtained by BX51M, Olympus. Fourier transform infrared (FT-IR) spectra were recorded using a Spectrum One system, Perkin-Elmer, with a wavenumber range of 500–4000 cm^−1^.

### 2.4. Measurement

The fabricated sensor was connected with wire using conducting tape for electrical measurements. Then, it was inserted in a humidity and temperature chamber (KCL-1000, EYELA) for testing. The capacitive responses of the sensor were measured as a function of RH with different ranges from 45% to 90% by a CV analyzer (4284A LCR meter, Agilent, Santa Clara, CA, USA) at 40 kHz. The sensor exposure time to each RH environment was 5 min. After measurements, the sensors were stored in a dry environment. Cyclic bending was performed using a multi-modal bending tester (ND-BTS01, Covotech, Hwaseong, Korea) with a controlled radius and number of bending cycles. After bending, the sensing characteristics were measured with re-flat condition.

For real-time monitoring, anisotropic conductive film bonder (ACF bonder, Digitech systems, Paju, Korea) were utilized to achieve electrical contact between the H-PTFE sensor and readout circuit. We already have mobile application systems used in previous studies. The humidity data of the H-PTFE sensor, transmitted via Bluetooth communication, was displayed in a mobile application.

## 3. Results and Discussion

**Figure 1:**
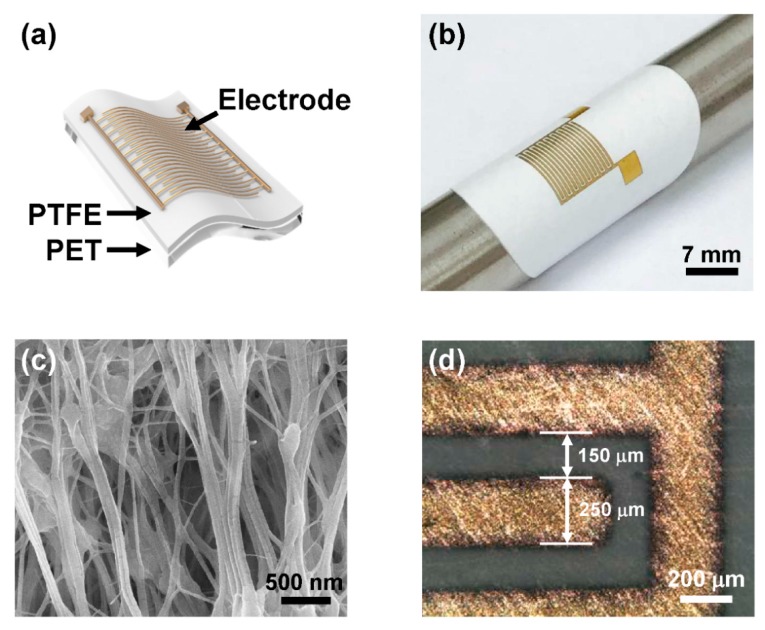
(**a**) 3D schematic structure and (**b**) photograph of a flexible H-PTFE humidity sensor; (**c**) FE-SEM image of the H-PTFE membrane; (**d**) Optical microscope image of the interdigitated electrode.

The schematic structure and a photograph of the H-PTFE flexible humidity sensor are shown in Figure 1a,b. The titanium/gold electrode was built onto the surface of the H-PTFE film, and the other side of the sensor was surrounded by PET film. As shown in Figure 1b, the proposed H-PTFE sensor has high flexibility, and is capable of measuring the humidity condition of curved materials. The H-PTFE membrane, which plays a pivotal role in humidity sensing, has an inherent ability for rapid water absorption and evaporation. This ability was induced by high surface-to-volume ratio from the random porous structure, as confirmed in the FE-SEM image shown in Figure 1c. The average pore size of the films is 0.45 µm and the overall porosity is 80%. Considering the requirements of good accuracy within wide ranges of humidity as well as easy fabrication, we designed an interdigitated humidity sensor device. It has also been noted that the interdigitated type shows good sensitivity compared with the parallel-plate type [18,19]. With the increase in the number of interdigitated fingers, the variation in capacitance value would have an increasingly wide range. However, the sensor with eight fingers is appropriate when both the sensing performance and device size are considered. In Figure 1d, the finger width of the sensor was found to be 250 µm, and the finger-to-finger distance was 150 µm.

Although H-PTFE plays a significant role in water absorption, the water affinity of the sensor can be enhanced by treatment of NaOH on the H-PTFE humidity sensor. Na^+^ ions have been reported to be very effective in changing the bond structure of PTFE [16,17]. The NaOH treatment process is performed when hydrophobic PTFE is transformed to H-PTFE [20]. When the device was immersed in NaOH solution, Na^+^ and OH^−^ ions infiltrated the H-PTFE pore structure. Then, the Na^+^ ions break the strong carbon (C)–fluorine (F) bonds, and produce a small quantity of C–OH bonds [20]. These molecular structures could enhance the water uptake sensitivity through the physical and chemical absorption of water molecules. 

**Figure 2:**
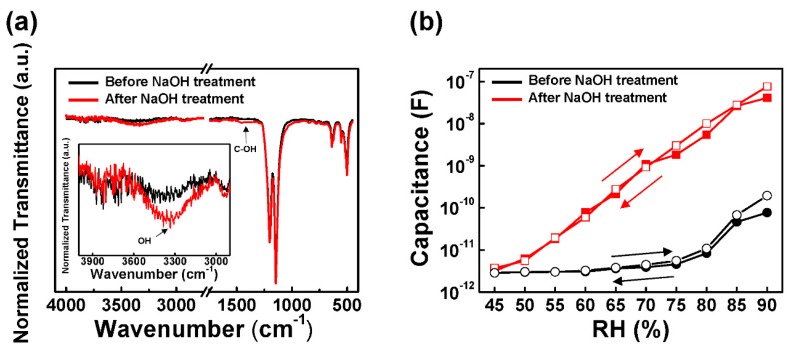
(**a**) FT-IR spectra of H-PTFE membrane with respect to NaOH treatment. Inset: 2900–4000 cm^−1^ region; (**b**) sensing response and hysteresis of H-PTFE flexible humidity sensor before and after NaOH treatment.

The FT-IR spectra provided information on the chemical functionalization of the PTFE surface upon treatment with NaOH. Figure 2a shows the adsorption spectra for the untreated and NaOH-treated H-PTFE films. The spectra of the original H-PTFE film showed the peaks of CF_2_ and CF_3_ groups at 1201 cm^−1^, 1146 cm^−1^, 638 cm^−1^_,_ and 502 cm^−1^ [21,22]. The additional generation of C–OH bonds on the surface of NaOH-treated H-PTFE was presented in a broad peak range of 1350–1500 cm^−1^ [23]. Also, as shown in the insets of Figure 2a, there is an increase in the peak area within the region of 3200–3500 cm^−^^1^ associated with OH [24]. This indicates that the reaction of NaOH with PTFE under aqueous conditions might have incorporated OH groups into the structures of PTFE. In this case, these OH groups could further adsorb moisture via hydrogen bonds. This demonstrates that the NaOH-treated surface can be used as a humidity sensor, even though further improvement should be required to satisfy the optimum conditions.

In Figure 2b, the humidity response characteristics of the H-PTFE sensors before and after adding NaOH solution were characterized with various RH, ranging from 45–90%. The temperature was maintained at 35 °C during all the measurements. The original H-PTFE humidity sensor without NaOH treatment has a value of 2.86 pF at the lowest RH of 45%. Until the RH reached 75%, notable capacitance variation was not revealed, with only a slight increase to 4.58 pF. The obvious increase was observed at over 75% RH, but the response curve shows nonlinear behavior. In the case of adding NaOH, the capacitance represented significantly more linear behavior and a much higher increase factor of 2 × 10^4^, compared to non-treated H-PTFE sensors under a same RH range. Not only was there the long-range linear behavior, but also the extremely low non-coincidence between absorption and desorption of the H-PTFE sensor with NaOH treatment was represented in Figure 2b. The curve with empty circles exhibits adsorption, and the curve with filled circles is the desorption of humidity. The maximum hysteresis of the H-PTFE humidity sensor was observed at an RH of 80%, but it can be considered negligible for interpreting the results.

Abundant OH groups in the NaOH-treated H-PTFE film promote the physical and chemical absorption of water molecules, which provides a great advantage in detecting humidity in low-RH regions. Also, in high-RH regions, the water molecules absorbed on the H-PTFE surface make a secondary layer through physical adsorption by adjacent water molecules, which demonstrates a large increase in capacitance responses [25].

**Figure 3:**
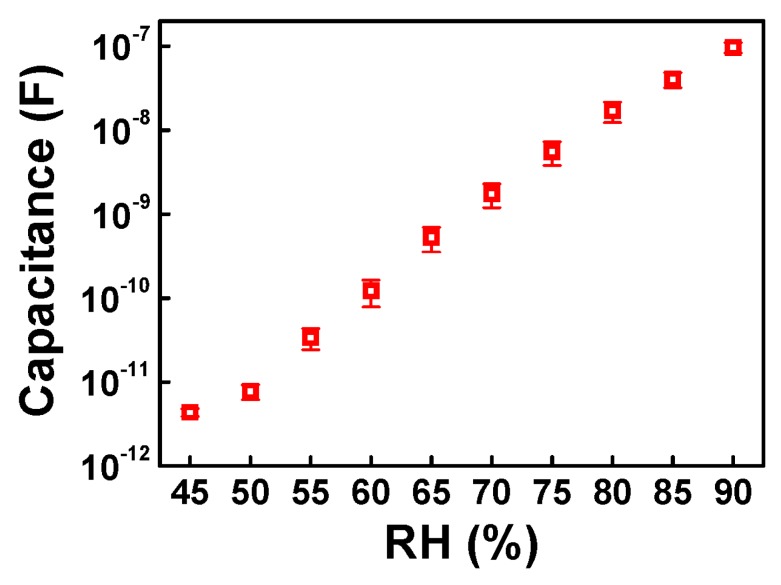
Average and standard error of sensing characteristics of four H-PTFE flexible humidity sensors.

To estimate the sensor performance in terms of reliability, we measured the capacitance responses of four H-PTFE sensors with regard to RH variation. Figure 3 reveals the average and standard error of the sensing responses of the H-PTFE sensors at each RH value. Each data point also shows linear behavior, and the standard errors are small, representing great linearity and reliability of the proposed sensor. These results imply that our sensor is substantially trustworthy for use in real-time monitoring. 

**Figure 4:**
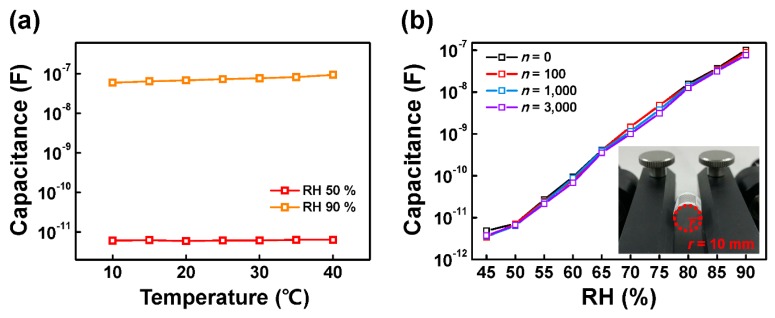
(**a**) Temperature-dependent characteristics of H-PTFE sensor with RH of 50% and 90%; (**b**) sensing behaviors of H-PTFE flexible humidity sensor under cyclic bending stress (*r* = 10 mm) with bending cycles of *n* = 0, 100, 1000, and 3000. Inset: image of cyclic bending test.

There are several requirements to use our H-PTFE humidity sensor for real-time monitoring. High sensitivity, linearity, and reliability have already been mentioned above, and in terms of stability, we conducted additional experiments. First, to verify the sensing stability of our sensor under different temperatures, sensing responses were measured under a temperature range from 10 °C to 40 °C. The RH was fixed at 50% and 90%, respectively. As shown in Figure 4a, the capacitance response of the curves at 50% RH and 90% RH increased slightly with increasing temperature. The curve of 90% RH shows more dependent characteristics on temperature than was seen at 50% RH. These results were understood as the increased thermal energy of water molecules. As the temperature increases, water molecules have strong thermal energy, which enhances the molecular polarization [26,27]. The large polarization is related to the increase in capacitance. Therefore, the temperature dependence must be calibrated to exclude the thermal effects.

Stable operation under mechanical stress also should be ensured for a flexible sensor. Herein, to confirm the performance variation of flexible H-PTFE humidity sensors under mechanical stress, we performed cyclic bending tests with different numbers of bending cycles (*n*) of 100, 1000, and 3000 with a 10 mm bending radius. The back of the H-PTFE film was laminated with a 70 µm thick PET film to downshift the mechanical stress. As shown in Figure 4b, the capacitance values slightly decrease with increasing numbers of bending cycles at most points. It should be noted that the flexible H-PTFE sensor has significant mechanical flexibility. Such high flexibility of the H-PTFE humidity sensor allows for a wide range of applications involving bendable and wearable sensors.

**Figure 5:**
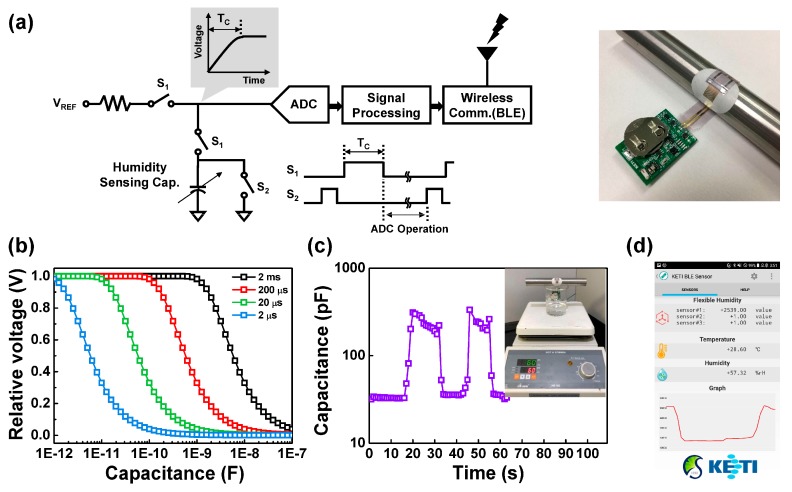
(**a**) Schematic layout of real-time monitoring system containing H-PTFE flexible humidity sensor, readout circuit, Bluetooth module. Inset: real-time monitoring system attached to a curved surface; (**b**) the wide ranges of capacitance at various switching timings (T_c_ = 2, 20, 200, and 2000 μs); (**c**) real-time monitoring data of water absorptions and evaporations when H-PTFE sensor was attached to a curved surface. Inset: setup condition for testing our real-time monitoring system; (**d**) mobile application.

To implement a real-time monitoring system with a flexible H-PTFE humidity sensor, an analog interface and a wireless communication using Bluetooth Low Energy (BLE) are designed, as shown in Figure 5a. The measured capacitance of the humidity sensor showed the properties of the exponentially wide-range variations. To process the wide-range capacitance data, a novel analog interface circuit was utilized. An RC network consisting of a 500-kΩ resistor and the humidity capacitance were utilized because the network has an inverse exponential characteristic. Thus, a linear voltage level corresponding to the humidity can be obtained when the switching timing of the network is controlled carefully. The wide capacitance range (from a few pF to hundreds of nF) can also be obtained with varying switching timing, as shown in Figure 5b. The following signal-processing block can calculate the overall humidity data based on the weighting factor of the switching time.

The flexible H-PTFE sensor was connected with the readout circuit by utilizing anisotropic conductive film bonder which is widely used for contact between electrodes. As shown in inset of Figure 5a, users can apply our real-time monitoring system anywhere with no restrictions because of its high flexibility and light weight. To further evaluate the real-time monitoring of the flexible H-PTFE sensor system, a testing situation was constructed using a beaker with hot water (60 °C), as shown in the inset of Figure 5c, where water vapor occurred above the beaker during experiment. The H-PTFE-based real-time monitoring sensor system attached to the curved surface was placed on the beaker in a few seconds and removed. The measured data is wirelessly transmitted to the mobile, which provides the information for humidity values as shown in Figure 5d. The capacitance value increased with exposure of water vapors and after removing, it decreased. The water absorption and release were precisely monitored using our sensor system. Based on these experimental findings, the proposed flexible humidity sensor device demonstrates improvement on potential applications in many fields.

## 4. Conclusions

In conclusion, we demonstrated the potential of a flexible sensor using H-PTFE as a dielectric sensitive film to perform reliable humidity monitoring. We showed that the sensor response was dependent on the RH. It was confirmed that NaOH treatment of the H-PTFE film further improved the sensitivity, detection dynamic range, and reliability of water uptake. The cycling bending test also proved the high mechanical flexibility of the humidity sensor. Finally, in order to validate the applicability for real-time monitoring, the flexible humidity sensor, integrated with an analog interface and wireless communication, was attached on the curved surface, and then was used to detect humidity changes. Our future efforts will be focused on improving the sensitivity and reproducibility of the sensor, and further extending this platform to humidity detection for industrial, environmental, and medical fields.
